# Simultaneous Ligand and Receptor Tracking through NMR Spectroscopy Enabled by Distinct ^19^F Labels

**DOI:** 10.3390/ijms20153658

**Published:** 2019-07-26

**Authors:** Jeffrey R. Simmons, Alexandre Murza, Michael D. Lumsden, Calem Kenward, Éric Marsault, Jan K. Rainey

**Affiliations:** 1 Department of Biochemistry & Molecular Biology, Dalhousie University, Halifax, NS B3H 4R2, Canada; 2 Institut de Pharmacologie, Université de Sherbrooke, Sherbrooke, QC J1H 5N4, Canada; 3 Department of Pharmacology-Physiology, Université de Sherbrooke, Sherbrooke, QC J1H 5N4, Canada; 4 Department of Chemistry, Dalhousie University, Halifax, NS B3H 4R2, Canada

**Keywords:** ^19^F NMR, G protein-coupled receptor (GPCR), apelin receptor (APJ), ligand-receptor interactions, peptide-GPCR binding, ^19^F-labelled apelin analogues

## Abstract

To probe ligand-receptor binding at the atomic-level, a frequent approach involves multidimensional nuclear magnetic resonance (NMR) spectroscopy experiments relying on ^13^C- and/or ^15^N-enrichment alongside ^1^H. Alternatively, the lack of fluorine in biomolecules may be exploited through specific incorporation of ^19^F nuclei into a sample. The ^19^F nucleus is highly sensitive to environmental changes and allows for one-dimensional NMR spectroscopic study, with perturbation to chemical shift and spin dynamics diagnostic of structural change, ligand binding, and modified conformational sampling. This was applied to the apelinergic system, which comprises a rhodopsin-like G protein-coupled receptor (the apelin receptor (AR)/APJ) and two families of cognate ligands, the apelin and apela (ELABELA/toddler) peptides. Specifically, AR fragments consisting of either the N-terminal tail and first transmembrane (TM) α-helix (AR55) or the first three transmembrane α-helices (TM1-3) were prepared with biosynthetic fluorotryptophan incorporation. Interactions of each AR fragment with a high-affinity, 2,4,5-trifluorophenylalanine labeled apelin analogue were compared by ^19^F NMR. Distinct ranges of ^19^F chemical shifts for ligand and receptor provide unambiguous tracking of both species, with distinct binding behaviour observed for each AR fragment implying that AR55 is not sufficient to recapitulate the physiological binding event. Site-specific perturbation was also apparent for the apelin analogue as a function of substitution site, indicating an orientational binding preference. As a whole, this strategy of distinctive ^19^F labelling for ligand and receptor provides a relatively fast (i.e., employing 1D NMR experiments) and highly sensitive method to simultaneously and definitively track binding in both species.

## 1. Introduction

The apelinergic system has been implicated in wide-ranging physiological processes, including vasodilation, cardiovascular development, and insulin homeostasis [1,2]. Correspondingly, this system is also involved in disease pathologies such as diabetes and chronic heart failure. The apelinergic system comprises a class A rhodopsin-like G protein-coupled receptor, the apelin receptor (AR; formerly APJ [3]), and two families of peptide ligand: apelin [4] and apela [5,6].

Apelin is a peptide expressed in peripheral tissue and the central nervous system, which regulates cardiac contractility through binding to AR [7]. Apela’s link to the AR was discovered relatively recently, acting to form an essential signal pathway for embryonic cardiovascular development [5]. Both peptides are secreted by cells as pre-proteins (77 and 54 residues long for apelin [4] and apela [5], respectively). Its final bioactive forms are 55, 36, 17, or 13 residues long for apelin [8,9] and 32, 22, or 11 residues long for apela [10]. In each case, the natural bioactive isoforms are formed by N-terminal truncations of the pre-protein, with the C-terminal 13 or 11 residues constitutively retained in the case of apelin and apela, respectively. In this work, we focus strictly on apelin-AR interactions.

Mechanistic understanding of the behaviour of these different bioactive ligand isoforms relies upon delineation of regions important for both binding to and activation of the receptor. From a biophysical standpoint, the “RPRL” motif in apelin (found at the N-terminal of the apelin-13 isoform) is relatively conformationally restrained in buffer regardless of isoform [9,11]. This motif shows both involvement in and increased structuring upon binding to membrane-mimetic micelles [12,13]. Although this phenomenon has not been probed at the atomic-level in a bilayer setting, bicelle binding by apelin has been demonstrated from the perspective of the phospholipid headgroup [14], with likely extension to cell membrane interactive behaviour. The apelin C-terminus (of sequence GPMPF), conversely, becomes structurally converged both when bound to micelles [12,13] and at low temperature [9,11].

**Figure 1:**
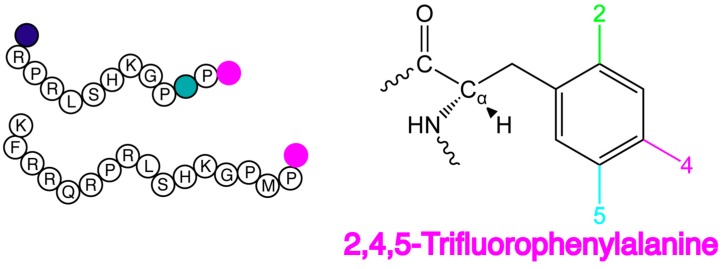
Amino acid sequence of F*-ap-13 (top) and F*-ap-17 (bottom), with the trifluorophenylalanine (right). Colored boxes indicate the non-standard amino acids pyroglutamate (blue), norleucine (teal) and the 2,4,5-trifluorophenylalanine label (pink).

Structure-activity relationships also directly implicate both the RPRL motif and apelin C-terminal segment as being functionally critical. A systematic alanine scan performed on apelin-13 showed that R2, R4 and F13 mutations led to important loss in binding [15]. The truncation of F17 in apelin-17 abrogated β-arrestin recruitment while maintaining cAMP inhibition, Ca^2+^ mobilization and cardiac actions in rat and human [15,16,17]. Lee et al. also confirmed that I.V. administration of the F13A mutant to rats antagonizes the hypotensive effect of apelin-13 [18]. Furthermore, a series of C-terminal substitutions by unnatural amino acids also showed that this residue is pivotal to improve both binding and signalling of the ligand compared to that of the endogenous peptide [19,20]. Of particular interest to this study, addition of electron-withdrawing groups to the phenyl ring, with 2,4,5-trifluorophenylalanine (2,4,5F-Phe), was found to increase affinity [20]. The C-terminally modified apelin analogue in question, compound 21 in the prior study [20], is employed herein (referred to as F*-ap-13, Figure 1). For direct comparison, based on extensive biophysical characterization [9,11,12,14], an apelin-17 analogue with a C-terminal 2,4,5F-Phe is also employed herein (referred to as F*-ap-17, Figure 1).

Understanding how apelinergic ligands are recognized by, bind to, and activate their receptor, alongside changes in both structure and dynamics that occur upon interaction, is crucial to understanding how this system functions. Both triple-resonance (i.e., ^1^H, ^15^N, and ^13^C) nuclear magnetic resonance (NMR) spectroscopy [21,22] and X-ray crystallography [23] have been applied to the apelin receptor. Both structure and dynamics of receptor fragments have been characterized by NMR spectroscopy. X-ray crystallography yielded a high-resolution structure of the full-length receptor in an inactive-like state bound to an apelin analogue agonist. While both techniques provide potential for outstanding atomic-resolution detail, tracking changes occurring upon ligand binding may be more straightforward through other approaches.

One notable alternative is ^19^F NMR spectroscopy [24]. This approach has many advantages over standard triple-resonance NMR characterization, such as decreased experimental time when compared to multi-dimensional triple-resonance experiments. This can be a crucial factor if the protein system is not stable for extended acquisition periods. ^19^F NMR is also highly sensitive, with a general lack of fluorine in natural biological systems leading to spectra devoid of background signals from non-target molecules. This has been taken advantage of in the past for many diverse applications by other research groups, such as pioneering in-cell NMR studies with intact platelets [25] and examination of changes in structure, dynamics and conformational equilibria for G protein-coupled receptors (GPCRs) [26,27,28,29,30,31,32].

**Figure 2:**
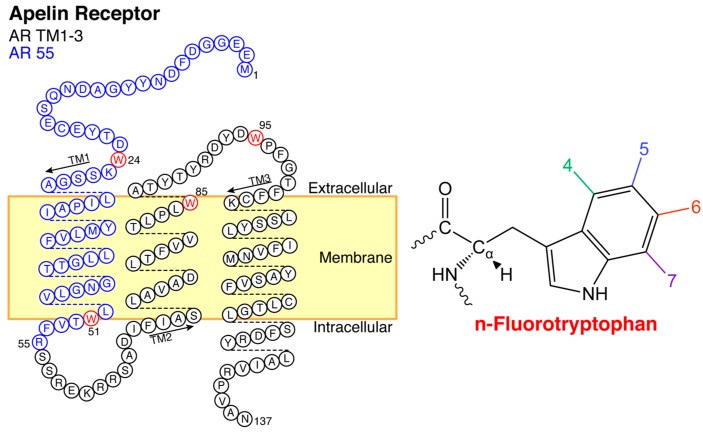
Biosynthetically ^19^F-labelled apelin receptor segments used as model systems to probe ligand binding interactions. Blue indicates AR55, black indicates TM1-3, and red indicates a tryptophan, with each possible fluorinated substituent numbered as shown on the right.

We have previously directly applied ^19^F NMR spectroscopy to study ligand binding to apelin receptor fragments [33]. In these prior studies, we took advantage of the ability to biosynthetically incorporate fluorine-labeled Trp (F-Trp) residues [34]. The low prevalence of Trp means that ^19^F labels are inherently relatively sparse, allowing for straightforward monitoring of well-defined sites. Through site-directed mutagenesis, signal assignment of four Trp residues covering the first three transmembrane (TM) segments of the AR was readily possible [33]. Specifically, two constructs were examined (Figure 2): (1) AR55, comprising residues 1-55 of the AR including the N-terminal tail and first TM segment [21]; and, (2) the TM1-3 fragment, comprising residues 1-138 covering the first 3 TM segments and N-terminal tail [35]. Using these receptor fragments labeled with 4F-, 5F-, 6F-, and 7F-Trp (Figure 2) at multiple locations, ^19^F NMR spectroscopy exhibited ligand-specific modulation of ^19^F NMR signal intensity at defined positions.

Here, we test the potential to extend this methodology through application of a ^19^F label with distinct chemical shift(s) from F-Trp residues (i.e., a spectroscopically “orthogonal” probe) to simultaneously monitor both ligand and receptor during investigations of binding interactions. This relies strictly upon 1D ^19^F NMR experiments, providing relatively rapid, highly sensitive, and fully site-specific characterization simultaneously for both ligand and receptor. We demonstrate this using the F*-ap-13 ligand with a C-terminal 2,4,5F-Phe residue characterized in the presence of both AR55 and TM1-3 labelled with various F-Trp types and solubilized in membrane-mimetic dodecylphosphocholine (DPC) or sodium dodecyl sulfate (SDS) micelles. The generality of 2,4,5F-Phe behaviour as an NMR probe is tested through comparison to the similarly C-terminally-modified F*-ap-17 peptide.

## 2. Results

### 2.1. F NMR Spectroscopic Behaviour of 2,4,5F-Phe

**Figure 3:**
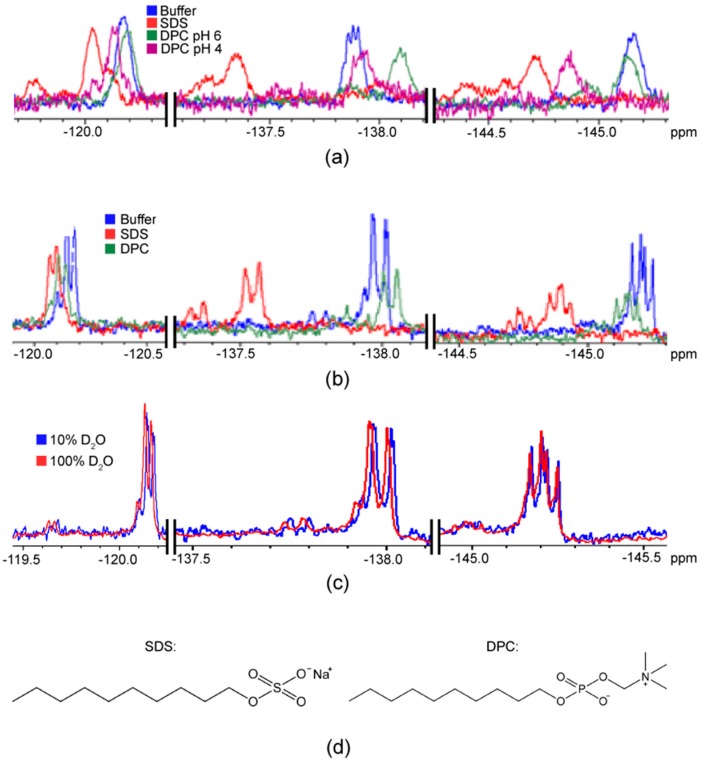
^19^F nuclear magnetic resonance (NMR) spectra of (**a**) F*-ap-13 and (**b**) F*-ap-17 under indicated experimental conditions. (**c**) F*-ap-17 in buffer with 10% D_2_O:90% H_2_O vs. 100% D_2_O. (**d**) Sodium dodecyl sulfate (SDS) and dodecylphosphocholine (DPC) structures.

Two distinct peptides with a C-terminal 2,4,5F-Phe were compared: F*-ap-13 and F*-ap-17. F*-ap-13 exhibited three distinct ^19^F chemical shifts at ~118.8, −136.7 and −145.2 ppm, while F*-ap-17 exhibited similar chemical shifts, with the ^19^F substituents at positions 4 and 5 being slightly more shielded than their F*-ap-13 counterparts (Figure 3a,b). The ^19^F substituents on F*-ap-17 exhibit a slightly shielded chemical shift in 100% D_2_O relative to 90% H_2_O/10% D_2_O (Figure 3c), consistent with a secondary isotope shift (SIS) [36].

**Figure 4:**
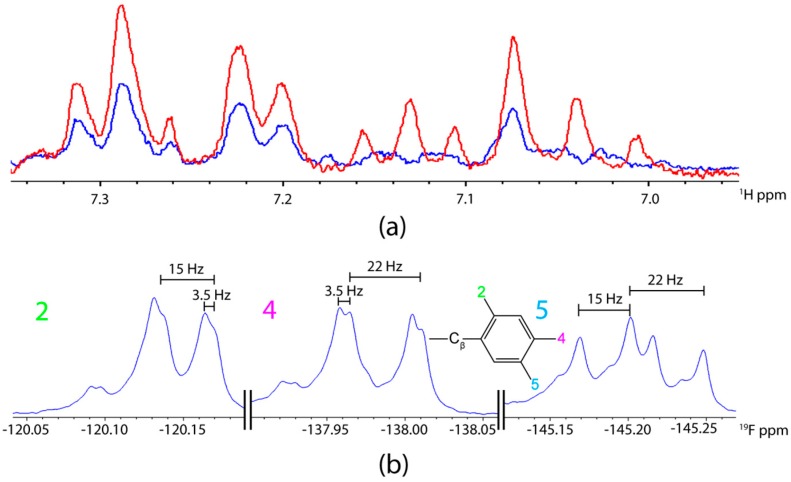
Assignment of 2,4,5-trifluorophenylalanine ^19^F resonances based on (**a**) ^1^H NMR with (red) and without (blue) selective decoupling of ^19^F at ~−145.2 ppm and (**b**) ^19^F-NMR J-coupling values with numbers 2, 4, and 5 corresponding to the 2,4,5-trifluorophenylalanine ring structure (inset). F*-ap-17 was employed in 100% D_2_O.

The three observed ^19^F resonances were readily assigned to the fluorine-substituents on the Phe side-chain through examination of selectively ^19^F-decoupled ^1^H NMR experiments. The two phenyl protons were first identified via an ^1^H{^19^F} experiment and were found to have chemical shifts of 7.15 and 7.05 ppm. Subsequently, a series of three different ^1^H NMR experiments were performed on F*-ap-17 involving the application of selective continuous-wave decoupling irradiation separately at each of the three ^19^F resonance positions. Only one of these experiments (decoupling at −145.2 ppm) resulted in triplet like patterns for both phenyl protons. These two triplets arise due to equal J-coupling to fluorine 2 and 4 in both cases; one triplet is due to ^3^J(^1^H–^19^F) = 9 Hz and another due to ^4^J(^1^H–^19^F) = 7 Hz (Figure 4a). Only the removal of the ^19^F J-coupling from the 5F-substituent is expected to result in triplet patterns for both protons; the other two decoupling experiments, as expected, resulted in doublet of doublet patterns. Therefore, these decoupling experiments allow definitive assignment of the ^19^F resonance at −145.2 ppm to the fluorine at the 5-position (Figure 4a).

With the 5F-substituent assigned, the 4F-substituent was readily attributable to the resonance at −136.7 ppm as this resonance showed a ~22 Hz coupling due to ^3^J(4F–F), whereas the −118.8 ppm resonance did not. Therefore, the latter signal is assigned to the 2F-substituent. It is worth noting that, based on these assignments, we can determine that 4J(2F–4F) = 3.5 Hz and 5J(2F–5F) = 15 Hz. The fact that 5J > 4J follows expectations for aromatics with ^19^F substituents [37].

Next, the effect of micellar constituents upon the ^19^F NMR spectroscopic behaviour of the C-terminal 2,4,5F-Phe of each apelin analogue was examined. Similar patterns were observed for both F*-ap-13 and F*-ap-17, with the 4F-substituent most perturbed by environmental conditions, changing 0.5–0.8 ppm between the 100 mM SDS and DPC conditions at pH 6 (Figure 3a,b; detergent structures compared in Figure 3d). The 5F-substiutent was also affected by micellar conditions (0.3–0.5 ppm change), while the 2F-substitutent was essentially insensitive to environmental effects. Unsurprisingly the chemical shift perturbations were found to be pH sensitive, showing more than 0.2–0.3 ppm changes for the 4F- and 5F-substitutents and 0.1 ppm for the 2F-substitutent at pH 4 vs. pH 6. This perturbation may be due to a change in the exchange equilibrium between the bound vs. unbound state of a given apelin ligand with the detergent micelle. Alternatively, environmental effects due to the solvent environment and/or charge density of the micelle surface may be manifesting through chemical shift perturbation.

Additional ^19^F resonances due to slow exchange on the NMR timescale were also manifest for 2,4,5F-Phe of F*-ap-13 and F*-ap-17 in the presence of both SDS and DPC micelles. This was most pronounced for the 4F- and 5F-substituents, with the 2F-substituent also exhibiting some degree of slow exchange in all micellar conditions except DPC micelles at pH 6. In general, all ^19^F substituents of 2,4,5F-Phe exhibited a greater degree of slow exchange in SDS micelle than DPC micelle samples.

### 2.2. Titration of AR Fragments with F*-ap-13

Based upon the high affinity of F*-ap-13 for the AR [20], this analogue was employed for titration studies. F-Trp-labelled AR55 was solubilized in DPC micelles (pH 4, as determined previously to be required for effective solubilization of this fragment in DPC [21]), while F-Trp-labelled AR TM1-3 was solubilized in SDS micelles (pH 6 [35]). In each instance, 1D ^19^F NMR experiments were employed to monitor the state of the C-terminal 2,4,5F-Phe of the F*-ap-13 ligand simultaneously with the state of the Trp residues of the AR fragment in question.

**Figure 5:**
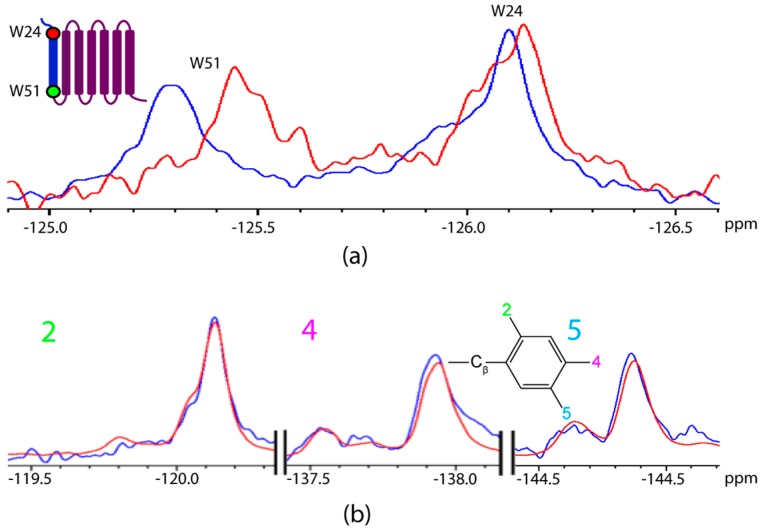
^19^F NMR spectral regions for (**a**) 4F-Trp-labelled AR55 (**b**) F*-ap-13. Experiments are compared for each species alone (blue) and at 1:1 ligand: receptor stoichiometry (red).

As 4F-Trp-labelled AR55 was titrated with F*-ap-13, the W51 site (on the intracellular face of the TM1 helix in the context of the full length receptor) became shielded by ~0.3 ppm, while the W24 site (in the extracellular N-terminal tail in the context of the full length receptor) exhibited a situation consistent with slow-exchange where new, slightly shielded and deshielded resonances are observed in convolution with the unliganded state. At ~equimolar stoichiometry, chemical shift perturbation was not observed for F*-ap-13 (Figure 5b; Table 1).

**Figure 6:**
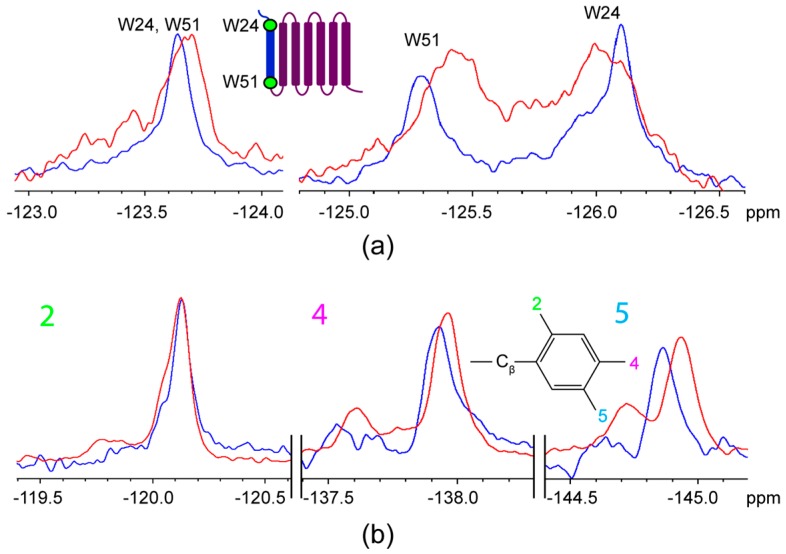
^19^F NMR spectral regions corresponding to (**a**) F-Trp labelled AR55 and (**b**) F*-ap-13. Experiments are compared for each species alone (blue) and at excess AR55 (red; ~4:1 AR55:F*-ap-13; an equimolar mixture of AR55 labelled with 4F-Trp (~125 ppm) and 6F-Trp (~123.7 ppm)).

At a stoichiometry of ~4:1 AR55:F*-ap-13, achieved through combination of both 4F-Trp and 6F-Trp AR55, W51 was disturbed similarly to the equimolar situation (Figure 6a; Table 1). W24, conversely, was perturbed to a greater degree than the equimolar situation, exhibiting a deshielded resonance with similar magnitude of Δδ to that of W51 (Figure 6a; Table 1). The slow-exchange phenomenon for W24 is still apparent, with significant intensity remaining consistent with the unliganded state but no further observation of a shielded conformation. At this stoichiometry, the F*-ap-13 ligand exhibited chemical shift perturbation at the 4F- and 5F-Phe substituents, with both being increasingly shielded and the 5F-substituent being more perturbed than the 4F-substiutent (Figure 6b; Table 1).

**Figure 7:**
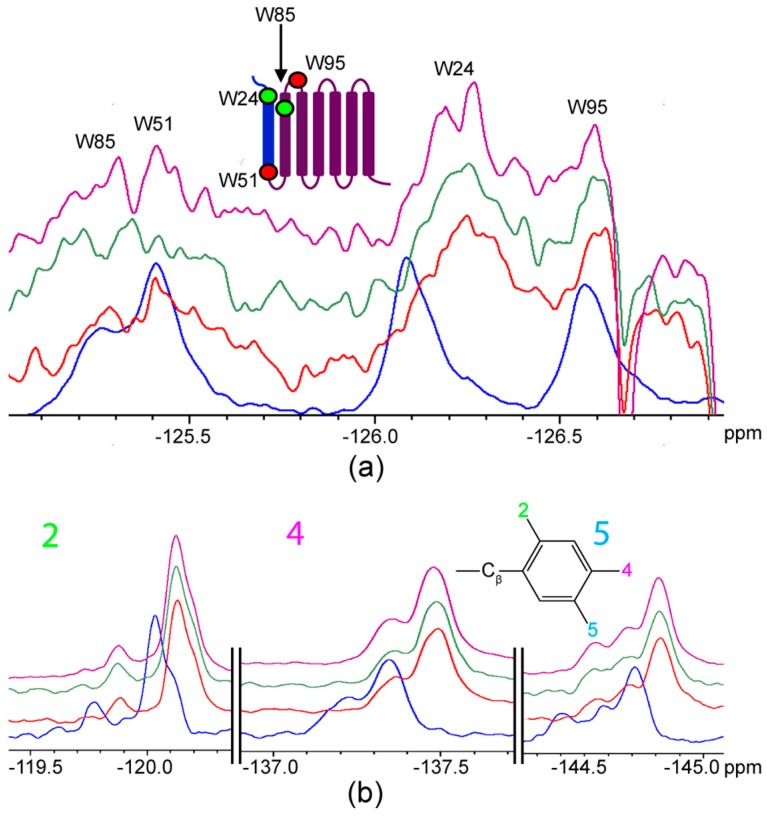
^19^F NMR spectra showing regions corresponding to (**a**) 4F-Trp-labelled TM1-3 and (**b**) F*-ap-13. Spectra are compared for each species alone (blue); at 1:2 ligand: receptor stoichiometry (red); 1:1 stoichiometry (green); and, 2:1 ligand:receptor stoichiometry (purple).

The behaviour of AR TM1-3 contrasted with AR55 in that the sites W24 and W85 (both on the extracellular face in the context of the full-length receptor) exhibited the most pronounced chemical shift perturbation as the ratio of ligand to receptor was elevated (Figure 7a; Table 2). Corresponding chemical shift perturbations were observed in all ligand fluorination sites (Figure 7b; Table 2), with the greatest perturbation observed at the 4F-Phe substituent. Interestingly, at approximately equimolar stoichiometry, W51 and W85 exhibited perturbation to a state that was not maintained at either excess ligand or excess receptor fragment (Figure 7a; Table 2).

## 3. Discussion

**Figure 8:**
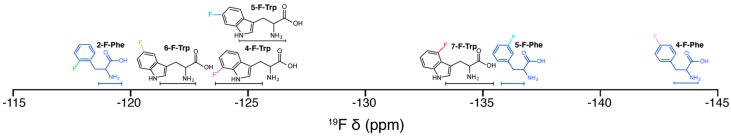
Observed chemical shift ranges for F substituents on 2,4,5-trifluorophenylalanine in F*-ap-13 and 17 (blue) and for each indicated F-Trp for AR55 and TM1-3 [33], and for 5F-Trp more generally [34,40].

The ^19^F chemical shift ranges observed for the 2,4,5F-Phe in buffer, micelles, and in titration with AR fragments (Figure 8), appear generally distinct from those of any of the F-Trp species routinely biosynthetically incorporated [33,34,38]. It should be noted that F-Trp chemical shifts may vary further than what has been observed in AR fragments [39], thus some optimization may be required to ensure that the class of F-Trp labeling used does not overlap in ^19^F chemical shift with 2,4,5F-Phe. This must also be balanced with the fact that, as we have noted previously [33], different F-Trp types give rise to varying degrees of chemical shift overlap or dispersion at a given Trp site in the protein. Here, no overlap was encountered between ligand and receptor in any case, providing clear and unambiguous tracking of the effects of titration upon both species.

From the ligand perspective, the substitution site-dependent chemical shifts of the 2,4,5-F-Phe probe provide an excellent opportunity with respect to the monitoring of both localization of and orientational preference of interactions. This was clear in both distinct chemical shift perturbation and evidence for slow exchange on the NMR timescale (i.e., the modulation of a given ^19^F resonance from a single peak to multiple peaks) upon interaction of F*-ap-13 and F*-ap-17 with both DPC and SDS micelles (Figure 3). Both phenomena were particularly accentuated for the 4F- and 5F-substituents relative to the 2F-substituent, consistent with the greater degree of accessibility of the 4F- and 5F-substituents. The 2F-substituent, in turn, thus would serve as an excellent “internal control” for a binding interaction. As an example, a shift in equilibrium towards favoring of a bound state may lead to changes in dynamics at the 2F-substiutent that are manifest in linewidth while the substituent remains relatively protected with respect to chemical shift perturbation.

It should be noted that the observed relatively modest increase in ^19^F resonance linewidth for the 2F substituent (Figure 3) is consistent with our prior observation of fast exchange for various apelin [12,13] and apela [10] species interacting with micelles, or even with slower-tumbling bicelles [14]. For future application, one may envision the 2F substituent—in particular—as a highly valuable probe to distinguish between a transient surface interaction vs. burial of the ligand C-terminus into a binding pocket, given the likelihood that the latter scenario would perturb the chemical shift at this site. Notably, this would potentially be highly valuable in the case of apelin, where the C-terminus has been proposed to penetrate quite deeply into the AR TM bundle [41]. The degree of penetration of the AMG3054 analogue was observed to be somewhat more modest in the recent AR crystal structure [23], an interesting distinction for future study.

Our development and characterization of the AR55 [21,22] and TM1-3 [35] fragments follows from the “divide and conquer” approach for membrane proteins [42]. In this approach, it is argued that individual TM segments of α-helical membrane proteins fold independently, recapitulating in isolation their structure in the context of the full-length protein. This structural recapitulation has indeed been frequently, although not always, observed when comparing fragment and full-length membrane protein structures [42]. Lending further credence to this, with AR55 we also found structuring of the TM1 segment to be robust, regardless of micellar environment [22], and the kink in the TM helix that we observed [21] is consistent with the AR crystal structure [23].

Clearly, ligand binding is likely to be a distinct situation that is far more reliant on tertiary structuring of a protein rather than strictly reliant upon correct secondary structuring and folding of dissected TM segments. The disparate titration behaviour observed for the AR55 (Figure 5 and Figure 6) vs. TM1-3 (Figure 7) fragments is quite informative from this regard. Namely, the interaction of F*-ap-13 with AR55 is most prevalently seen at the W51 site. This is entirely physiologically unreasonable given the topology in the context of the full-length GPCR, where W51 is located at the intracellular face of TM1. In the AR55 context, the slow-exchange behaviour observed for W24, with distinct equilibria apparent at 1:1 vs. 1:4 ligand:receptor ratios may arise from some form of conformational selection induced by ligand interaction [32]. However, further experimental characterization is required to fully characterize this phenomenon.

In TM1-3, the primary ^19^F NMR perturbations are observed at W24 and W85, located on the extracellular face in the context of the full-length GPCR. W24, in particular, is proximal to anionic residues noted through mutagenesis to be involved in ligand binding [21,43]. Correspondingly, one crystallographically-observed site of extracellular interaction for the apelin analogue AMG3054 with the AR occurs in this region [23]. This may imply that the TM1-3 fragment is sufficient to bias ligand binding toward the correct face (i.e., the extracellular side), whereas the AR55 fragment is not with significant W24 signal consistently observed in the unliganded-state. Alternatively, the first intracellular loop containing W51 may be structured in such a way in the TM1-3 fragment that F*-ap-13 binding is less favored than it is in the unencumbered AR55 context. This latter hypothesis seems less plausible than the former, given the observation of W51 perturbation at an intermediate point in the TM1-3:F*-ap-13 titration (Figure 7).

Comparing the TM1-3:F*-ap-13 interaction to our prior studies, titration of AR TM1-3 with apelin-36 led to ^19^F signal attenuation most prevalently at the W95 site without observable chemical shift perturbation at any of the four Trp residues in TM1-3 [33]. This is in distinct contrast to the present situation with F*-ap-13, where chemical shift perturbation was clear for both W24 and W85. However, a similar propensity for binding to the extracellular portion of the GPCR is retained for both ligands and the TM1-3 fragment of AR. Given the variation in bioactivity of apelin isoforms as a function of length (reviewed in [2]), as well as isoform-dependent differences in affinity [2,9], isoform-dependent receptor-binding mechanisms that manifest in ^19^F NMR behaviour may not be unexpected. However, a more in-depth and systematic comparison of ligand-receptor interactions in the apelinergic system both with respect to screening a greater variety of ligands and employing an AR construct with at least an intact 7-TM bundle, if not the full-length GPCR, is required to comprehensively understand the intriguing distinctions in bioactivity provided by a series of highly similar ligands activating a single GPCR.

In summary, spectroscopically-orthogonal ^19^F probes on ligand and receptor provide an outstanding means of probing binding with exquisite molecular specificity, site-specificity within a given molecule, and high sensitivity. This would be equally valuable in, e.g., probing of enzymatic interactions, or in any situation where characterization of intermolecular interactions might be confounded by overlap of chemical shifts between species. Solution-state ^19^F NMR spectroscopy may notably be applied even in situations with relatively high molecular weight/slow tumbling (e.g., a recent comprehensive enzyme kinetics characterization of a ~90 kDa Hsp90 enabled by ^19^F NMR methods [44] or demonstration of a new ^19^F-13C transverse relaxation optimized spectroscopy (TROSY) NMR methodology capable of reporting on a ~180 kDa α7 single-ring of a 20S proteasome core particle [45]). This adds to the versatility and cost-effectiveness of this technique relative to, e.g., the requirement to deuterate the target protein and specifically introduce ^1^H at desired probe sites. The ability to simultaneously monitor both ligand and receptor by ^19^F NMR specifically allows for examination of: (1) whether binding takes place, (2) where binding occurs, and (3) whether binding depends upon the molecular species employed (e.g., disparate AR fragments, as herein, or disparate apelin or apela isoforms, moving forward). Beyond probing binding from a biophysical perspective this could be applied to disease pathology models, examining the manner in which regions deeper in the receptor are changed in binding during different disease states.

## 4. Materials and Methods

### 4.1. Apelin Receptor Fragments

Both apelin receptor fragments were prepared with biosynthetic fluorotryptophan incorporation, as previously reported [33]. Briefly, fluorine-labeled protein was expressed by BL21 (DE3) *Escherichia coli* in M9 medium [46] through induction with a solution of 120 mg/L of isopropyl β-d-1-thiogalactopyranoside and the desired fluoroindole in 1 mL dimethyl sulfoxide [34]. Following induction, cells were grown for 4 h at 37 °C, pelleted by centrifugation at 6500× *g* for 20 min at 4 °C, and lysed via French pressure cell (American Instrument Company, Silver Springs, MD, USA) and the receptor fragment in question was isolated from the inclusion body as detailed previously for AR55 [21,35]. Following reverse phase high performance liquid chromatography (RP-HPLC), as optimized for each receptor fragment [21,35], eluent fractions containing the target ^19^F-labelled protein were pooled and lyophilized prior to storage at −20 °C.

### 4.2. F*-ap-13 and F*-ap-17 Synthesis and Purification

High-affinity 2,4,5-trifluorophenylalanine-containing apelin analogues (referred to herein as F*-ap-13, specifically compound number 21 from [20]) and F*-ap-17 were prepared as described previously [20]. Briefly, peptides were synthesized using a TentaGel S PHB, O-[4-(hydroxymethyl)phenyl]polyethylene glycol resin (Rapp Polymere, Tübingen, Germany) with standard Fmoc-protection chemistry. Couplings were carried out using *O*-(7-azabenzotriazol-1-yl)-1,1,3,3-tetramethyluronium hexafluorophosphate (HATU, 5 equiv) and *N*,*N*-diisopropylethylamine (10 equiv) in *N*,*N*-dimethylformamide (DMF) for 1 h at room temperature, deprotections using piperidine (20% in DMF, 2 × 10 min at room temperature), and final cleavage from the resin using trifluoroacetic acid/H_2_O/triisopropylsilane/1,2-ethandithiol (37:1:1:1 v/v; 4 h at room temperature). Following precipitation in *tert*-butyl methyl ether at 0 °C, RP-HPLC was employed for purification, with peptide UV purity (>95%) and identity confirmed by electrospray ionization mass spectrometry. Purified peptides were lyophilized and stored at −20 °C.

### 4.3. 19F NMR Spectroscopy

All NMR samples were prepared in 20 mM sodium phosphate buffer (pH 6; adjusted using NaOH and HCl, as needed) containing 1 mM sodium azide, 0.5 mM 4,4-dimethyl-4-silapentane-1-sulfonic acid, and at 10% D_2_O/90% H_2_O unless specifically noted as 100% D_2_O. Samples of both F*-ap-13 and F*-ap-17 were prepared in this buffer without detergent and with the addition of either 100 mM DPC or 100 mM SDS. An F*-ap-13 sample in DPC was also adjusted to pH 4 to allow for direct comparison to the conditions required for AR55 solubilization. To probe ligand:receptor interactions, AR55 samples were solublized in 100 mM DPC (pH 4) while TM1-3 samples were solubilized in 100 mM SDS (pH 6). Ligand:receptor samples at different stoichiometries were prepared through titration for AR55. Conversely, samples at 1:2, 1:1 and 2:1 receptor:ligand stoichiometries were prepared individually for TM1-3. For 2,4,5F-Phe ^19^F resonance assignment purposes, 2,4-difluoronitrobenzene (Sigma; St. Louis, MO, USA) was employed as a model, two fluorine containing aromatic compound to ensure that soft decoupling power was suitable for complete but selective decoupling.

All samples were prepared in 5 mm susceptibility-matched Shigemi tubes (BMS-005B, Shigemi, Alliston, PA, USA). 1D ^1^H experiments with selective ^19^F decoupling at −145.2 ppm (with exact decoupler offset finetuned) were acquired using a 7.05 T Avance NMR spectrometer equipped with a 5 mm BBFO probe (Bruker Canada, Milton, ON, Canada) at 37 °C. All other 1D ^19^F NMR experiments were conducted using an 11.7 T Avance NMR spectrometer equipped with a 5 mm BBFO SmartProbe (Bruker Canada, Milton, ON, Canada) at 37 °C. A sweepwidth of 100.25 ppm, a transmitter offset of −110 ppm, and a recycle delay of 1 s were uniformly used for 1D ^19^F NMR experiments; other experimental parameters are detailed in Table 3. After data acquisition, TopSpin 4.0 (Bruker) was employed for processing and analysis with exponential line broadening applied (0.25 Hz for ^1^H NMR experiments; 2 Hz for ^19^F NMR comparison of ligand in absence of receptor; 15 Hz for ^19^F experiments including AR55 or TM1-3 as well as ligand control experiments overlaid with the titration data) and manual baseline correction employed for all ^19^F NMR data. Data for AR55 and TM1-3 without ligand were previously reported [33], and reprocessed here for consistency to allow direct overlay and comparison with titration data.

## Figures and Tables

**Table 1:** ^19^F chemical shift perturbation (Δδ) relative to control sample in absence of binding partner for AR55:F*-ap-13 interactions.

		Δδ (ppm)	
Species	Location	1:1 ligand:receptor	1:4 ligand:receptor
4F-Trp AR55	W24	−0.03 ^a^	0.11
4F-Trp AR55	W51	−0.15–0.3 ^b^	−0.13
6F-Trp AR55	W24/W51	-	0.05 ^c^
F*-ap-13	2F on Phe	0	0.01
F*-ap-13	4F on Phe	−0.02	−0.04
F*-ap-13	5F on Phe	−0.01	−0.07

^a^ Δδ for the additional, shielded resonance. ^b^ Reported as the range of perturbation relative to the original peak maximum.^c^ Δδ determined using center of the two peaks that become apparent upon ligand addition (Figure 6a; red spectrum in the left panel) relative to unliganded form (Figure 6a; blue spectrum in the left panel) since unambiguous assignment of each to a given Trp is not possible.

**Table 2:** ^19^F chemical shift perturbation (Δδ) relative to control sample in absence of binding partner for TM1-3:F*-ap-13 interactions.

		Δδ (ppm)		
Species	Location	1:2 ligand:receptor	1:1 ligand:receptor	2:1 ligand:receptor
4F-Trp TM1-3	W24	−0.17	−0.16	−0.16
4F-Trp TM1-3	W51	0	0.07	0
4F-Trp TM1-3	W85	−0.02	0.06	−0.04
4F-Trp TM1-3	W95	−0.04	−0.04	−0.03
F*-ap-13	2F on Phe	−0.10	−0.09	−0.9
F*-ap-13	4F on Phe	0.14	0.14	0.13
F*-ap-13	5F on Phe	−0.11	−0.10	−0.10

**Table 3:** Sample conditions and experimental parameters employed for ^19^F and ^1^H NMR data collection.

Condition	Concentration(s)	Experiment	No. of Scans
^19^F NMR experiments			
F*-ap-13 no surfactant	F*-ap-13: 0.5 mM	zgflqn	384
F*-ap-13 with SDS	F*-ap-13: 0.5 mM	zgflqn	384
F*-ap-13 with DPC	F*-ap-13: 0.5 mM	zgflqn	384
F*-ap-17 no surfactant	F*-ap-17: 0.25 mM	Zgfhigqn ^a^	1024
F*-ap-17 with SDS	F*-ap-17: 0.25 mM	Zgfhigqn ^a^	1024
F*-ap-17 with DPC	F*-ap-17: 0.25 mM	Zgfhigqn ^a^	1024
F*-ap-17 in 100% D_2_O no surfactant	F*-ap-17: 0.25 mM	Zgfhigqn ^a^	1024
1:1 AR55:F*-ap-13	AR55 (4′): 0.5 mM F*-ap-13: 0.5 mM	Zgfhigqn ^a^	22,000
1:2 TM1-3: F*-ap-13	TM1-3 (4′): 0.05 mM F*-ap-13: 0.1 mM	Zgfhigqn ^a^	19,456
1:1 TM1-3: F*-ap-13	TM1-3 (4′): 0.1 mM F*-ap-13: 0.1 mM	Zgfhigqn ^a^	3072
2:1 TM1-3:F*-ap-13	TM1-3 (4′): 0.15 mM F*-ap-13: 0.1 mM	Zgfhigqn ^a^	3072
4:1 AR55:F*-ap-13	AR55 (4′): 0.25 mM AR55 (6′): 0.25 mM F*-ap-13: 0.125 mM	Zgfhigqn ^a^	24,576
^1^H NMR experiments			
F*-ap-17 in 100% D_2_O no surfactant	F*-ap-17: 0.25 mM	zg	16
F*-ap-17 Soft Decoupling (100% D_2_O, no surfactant, −135 ppm, pl24 of 25 dB)	F*-ap-17: 0.25 mM	In-house experiment ^b^	32

^a^ Following comparison of ^19^F NMR experimental sensitivity with and without ^1^H decoupling, ^1^H was consistently not employed for any data presented here (i.e., decoupler power d0 was set to 120 dB/0 W). ^b^ Pulse program available upon request.

## Abbreviations

AR	Apelin receptor (APJ)
AR55	apelin receptor fragment comprising N-terminal tail and first transmembrane segment
DPC	Dodecylphosphocholine
GPCR	G-protein-coupled receptor
NMR	Nuclear magnetic resonance
RP-HPLC	Reverse phase high performance liquid chromatography
SDS	Sodium dodecyl sulphate
TFA	Trifluoroacetic acid
TM	Transmembrane
TM1-3	apelin receptor fragment comprising N-terminal tail and first three transmembrane segments
